# Minding Animals: A Transdisciplinary Approach for Furthering Our Understanding of Animals in Society

**DOI:** 10.3390/ani1010004

**Published:** 2010-12-08

**Authors:** Marc Bekoff

**Affiliations:** Ecology and Evolutionary Biology, University of Colorado, Boulder, Colorado 80309, USA; E-Mail: marc.bekoff@gmail.com

I'm honored to be the guest editor of this volume of *Animals*. The essays included here are in the spirit of this new and forward-looking journal http://www.mdpi.com/2076-2615/1/1/1/pdf. They stem from a precedent setting gathering of scholars from all over the world representing many different disciplines at a meeting called ‘Minding Animals’, held in Newcastle, Australia in July 2009 (http://www.mindinganimals.com//index.php?option=com_content&task=view&id=210&Itemid=236). All of the delegates who journeyed from varying distances, sometimes huge, to be part of this unique gathering, shared a deep interest in learning more about who nonhuman animals (hereafter, animals) are from colleagues studying them from various perspectives, representing disciplines including biology, psychology, anthropology, and the social sciences and humanities. Not surprisingly, the meeting was characterized by great enthusiasm, lots of discussion often bordering on the frenetic since people would soon be dispersing to their homelands and not be readily accessible, and a commitment to continue learning more about animals in society.

The title of the conference, ‘Minding Animals’, was also the title of a book of mine [1]. ‘Minding animals’ means we must ‘mind’ them by recognizing that they have active minds and feelings. We must also ‘mind’ them as their caretakers in a human dominated world in which their interests are continually trumped in deference to ours. To mind animals it's essential for people with varied expertise and interests to talk to one another, to share what we know about animals and use this knowledge for bettering their and our lives. There are many ways of knowing and figuring out how science and the humanities, including those interested in animal protection, conservation, and environmentalism (with concerns ranging from individuals to populations, species, and ecosystems), can learn from one another is essential. This is just what happened in Newcastle.

The essays included here and other papers presented at the conference clearly showed that our relationships with nonhuman animals are complicated, frustrating, ambiguous, paradoxical, and range all over the place. When people tell me that they love animals and then harm or kill them I tell them I'm glad they don't love me. Surely we can do better in our relationships with animals and other people. Indeed, our relationships with human animals often are of the same ilk. We observe animals, gawk at them in wonder, experiment on them, eat them, wear them, write about them, draw and paint them, move them from here to there as we ‘redecorate nature,’ make decisions for them without their consent, and represent them in many varied ways, yet we often dispassionately ignore *who* they are and what they want and need. ‘Redecorating nature’ refers to the global tendency, almost a human obsession, to move into the living rooms of other animals with little or no regard for what we are doing to them, their friends, and their families [2,3,4]. We unrelentingly intrude because there are too many of us.

We still have a long way to go. Existing laws and regulations allow animals living on earth, in water, and in air to be treated in regrettable ways that demean us as a species. Indeed, in the eyes of the law animals are mere property and they can be treated like backpacks, couches, and bicycles with no legal recourse. The animals own eyes tell us that they don't like this at all. They do, of course, have a point of view. Objective views of animals don't work. We also double-cross animals. I can imagine an utterly exhausted polar bear asking, ‘Where's the ice?’ as she attempts to swim with her offspring from one ice floe to another as she had in years past only to discover that the ice is gone due to climate change. Despite global attempts to protect animals from wanton use and abuse, what we've been doing hasn't been working—‘good welfare’ just isn’t ‘good enough.’ Excuses justifying animal exploitation such as ‘Well, it's okay, I’m doing this in the name of science' or ‘in the name of this or that’ usually mean ‘in the name of humans.’ We're a very arrogant and self-centered lot.

A good way to make the world a more compassionate and peaceful place for all animals, to expand our compassion footprint, is to “mind” them ([4]; http://animals.change.org/blog/view/six_reasons_to_expand_your_compassion_footprint) It's time for people to begin to think about how to accrue compassion credits as they do carbon credits (see for example http://www.time.com/time/health/article/0,8599,1709186,00.html). Every individual can make positive changes for all living beings by weaving compassion, empathy, respect, dignity, peace, and love into their lives. It's simple to make more compassionate choices about what we eat and wear and how we educate students, conduct research, and entertain ourselves at animal's expenses. Increased compassion for animals can readily lead to less carbon because there's an inverse relationship between these markers especially in our consumption of factory-farmed meat from highly abused animals (http://www.ciwf.org.uk/globalwarning/index.html). We can also focus on the value of individual lives when we try to restore animal populations and ecosystems. It's fair to ask if the life of an individual should be traded off for the good of their species, for example, when we try to restore wolves to Yellowstone National Park and individual wolves die so that others might live?

It's a win-win situation to make every attempt to coexist peacefully and to do so in the most compassionate ways possible. For compassion for animals will make for more compassion among people and that's what we need as we journey into the future. Albert Schweitzer once wrote: “Until he extends his circle of compassion for all living things, man will not himself find peace.”

We can always add more compassion to the world. Ultimately, I believe compassion for animals will make for more compassion among people, weaving more empathy, respect, dignity, and love into all our lives. Animals are asking us to treat them better or leave them alone; that is their manifesto [4,5]. So, whenever you try to reduce carbon at the same time try to expand compassion. Animals and future generations of humans will thank us for our efforts and I'm sure each of us will feel better about ourselves. As the movie *Avatar* showed us, it's not all about us (http://animals.change.org/blog/view/iavatari_avarice_and_animals_its_not_all_about_us). We need to rewild our hearts and build corridors of compassion and co-existence that include all beings. We're not the only show in town.

The inaugural meeting was incredibly successful and many others and I look forward to the next gathering called ‘Minding Animals 2′—http://www.mindinganimals.com/.

## References

[1] Bekoff M. (2002). Minding Animals: Awareness, Emotions, and Heart.

[2] Bekoff M. (2006). Animal Passions and Beastly Virtues: Reflections on Redecorating Nature.

[3] Bekoff M. (2007). The Emotional Lives of Animals.

[4] Bekoff M. (2010). The Animal Manifesto: Six Reasons for Expanding Our Compassion Footprint.

[5] Bekoff M. http://animals.change.org/blog?author_id=609.

